# Information Based Diagnostic for Genetic Variance Parameter Estimation in Multi-Environment Trials

**DOI:** 10.3389/fpls.2021.785430

**Published:** 2021-12-07

**Authors:** Chris Lisle, Alison Smith, Carole L. Birrell, Brian Cullis

**Affiliations:** ^1^Centre for Biometrics and Data Science for Sustainable Primary Industries, School of Mathematics and Applied Statistics, National Institute for Applied Statistics Research Australia, University of Wollongong, Wollongong, NSW, Australia; ^2^School of Mathematics and Applied Statistics, National Institute for Applied Statistics Research Australia, University of Wollongong, Wollongong, NSW, Australia

**Keywords:** multi-environment trials, linear mixed models, D-optimality, variety connectivity, simulation study

## Abstract

Plant breeding programs evaluate varieties in series of field trials across years and locations, referred to as multi-environment trials (METs). These are an essential part of variety evaluation with the key aim of the statistical analysis of these datasets to accurately estimate the variety by environment (VE) effects. It has previously been thought that the number of varieties in common between environments, referred to as “variety connectivity,” was a key driver of the reliability of genetic variance parameter estimation and that this in turn affected the reliability of predictions of VE effects. In this paper we have provided the link between the objectives of this work and those in model-based experimental design. We propose the use of the D-optimality criterion as a diagnostic to capture the information available for the residual maximum likelihood (REML) estimation of the genetic variance parameters. We demonstrate the methods for a dataset with pedigree information as well as evaluating the performance of the diagnostic using two simulation studies. This measure is shown to provide a superior diagnostic to the traditional connectivity type measure in the sense of better forecasting the uncertainty of genetic variance parameter estimates.

## 1. Introduction

The objective of plant breeding is to breed superior plant varieties for various traits of economic importance. Selection of superior varieties is a result of the data analysis from a series of plant variety trials at a number of locations and possibly over several years, which are known as Multi-Environment Trials (METs). The breeding process is a progressive system involving the selection of superior varieties for further rounds of testing, removal of poor performing varieties and the inclusion of new varieties. This leads to datasets with varying levels of balance with respect to the number of varieties in common between environments, a measure known as “varietal connectivity.” Unbalanced datasets are common when the MET spans multiple breeding stages and seasons.

Given that most MET datasets are unbalanced and exhibit variance heterogeneity (Cullis et al., 2000; Chapman et al., 2003), it is critical that appropriate statistical methodology is used for analysis. Various authors have recommended the use of factor analytic (Smith et al., 2001b) linear mixed models (FALMM) to model the variety effects in different environments (VE effects), with a one-stage approach referred to as the gold standard of analysis (Gogel et al., 2018). The FALMM approach has been shown to provide a parsimonious and computationally stable approximation to the fully saturated unstructured model (Kelly et al., 2007). It is used in many plant breeding programs within Australia and overseas due to its flexibility and ability to handle unbalanced datasets. Additionally, it provides reliable predictions of the VE effects which can then be summarised in an informative manner using the selection tools of Smith and Cullis (2018) and Smith et al. (2021b).

Not only is it important to use an appropriate method of analysis for MET data, it is also crucial to construct a suitable dataset. The latter has only recently been addressed in the literature. Smith et al. (2021a) outlined an approach for constructing MET datasets that optimises the information available for selection decisions. This is based on new concepts that characterise the structure of a breeding program, defining groups of varieties that enter the initial testing stage of the breeding program in the same year to have come from the same “contemporary group” (CG). MET datasets are formed by combining bands of data to trace the selection histories of varieties within CG to maximise the amount of direct information. For a given dataset the A-optimality criterion from the model-based design literature is used to quantify the information for any given selection decision. The criterion is based on the A-value which is the average pairwise variance of elementary variety contrasts. This is appropriate for assessing variety effects because a small average pairwise variance is synonymous with a low probability of making incorrect selection decisions (Bueno Filho and Gilmour, 2003). A-values are computed using a specified linear mixed model (LMM), requiring the specification of the fixed and random effects and values for the variance parameters. The Smith et al. (2021a) approach assumes known variance parameters, whereas in practise they are required to be estimated. Hence, the purpose of this paper is to develop a diagnostic that measures the likely reliability of genetic variance parameter estimates for a given MET dataset. This could then be used in conjunction with Smith et al. (2021a).

It had previously been thought that variety connectivity was a key driver of the reliability of genetic variance parameter estimation and that this in turn affected the reliability of predictions of VE effects (Smith et al., 2001a, 2015; Ward et al., 2019). To combat these concerns, problematic environments were often removed from MET datasets if they appeared to have insufficient numbers of varieties in common with other environments. However, there has been little work to establish whether variety connectivity is the most appropriate measure to use for this purpose. Lisle (pers. comm) found that although variety connectivity was influential, there appeared to be other factors at play. Additionally, the number of varieties in common between environments may not be relevant for analyzes in which information on genetic relatedness is included since “connectivity” in this case is a more general concept.

In this paper we address the issue in a formal manner by considering the information for estimation of genetic variance parameters. As in Smith et al. (2021a) we use model-based experimental design concepts and assess information using a pre-specified LMM. In this setting our interest lies in the reliability of genetic variance parameter estimates across all environments, and for individual environments. We therefore consider the D-optimality criterion which measures the generalised variance of parameter estimates (Butler, 2013; Russell, 2018). This measure is typically used in the context of design searches for estimating (fixed) treatment effects (Atkinson et al., 2007), whereas the application to the problem of variance parameter estimation is not common in the literature. However, Loeza-Serrano and Donev (2014) and Nuga et al. (2017) considered D-optimality to search for experimental designs for estimating variance components. As in Smith et al. (2021a) our application does not involve a design search but rather the quantification of information for a given design (dataset).

In this paper we consider the impact of MET dataset structure on the reliability of residual maximum likelihood (REML) estimates of genetic variance parameters by proposing D-optimality values as a diagnostic measure. The paper is arranged as follows. Section 2 describes the statistical methodology, including a one-stage and two-stage approach for computing the diagnostic. The methods allow for the inclusion of information on genetic relatedness. In section 3, we apply the diagnostic to a durum wheat MET dataset, then examine the performance of the diagnostic using two simulation studies. Some concluding remarks are given in section 4.

## 2. Statistical Methods

### 2.1. Model for Analysis

Let ***y***_*j*_ denote the *n*_*j*_−vector of data for the *j*^*th*^ environment, *j* = 1, …, *p*. We then let ***y*** denote the *n*−vector of data combined across all environments in the MET, so write $y={\left(y_{1}^{⊤},y_{2}^{⊤},\ldots ,y_{p}^{⊤}\right)}^{⊤}$. Note that $n=\overset{p}{\underset{j=1}{\sum }}n_{j}$. The LMM for ***y*** can be written as:


(1) $$\begin{array}{l} y=X\tau +Z_{g}u_{g}+Z_{p}u_{p}+e \end{array}$$

where **τ** is a vector of fixed effects with associated design matrix ***X***; ***u***_***g***_ is the vector of random genetic effects with associated design matrix ***Z***_***g***_; ***u***_***p***_ is a vector of random non-genetic (or peripheral) effects with associated design matrix ***Z***_***p***_ and $e={\left(e_{1}^{⊤},e_{2}^{⊤},\ldots ,e_{p}^{⊤}\right)}^{⊤}$ is the combined vector of residuals from all environments. The vector of fixed effects includes mean parameters for individual environments. The vector of random peripheral effects includes effects associated with the experimental designs within environments. It is assumed:


(2) $$\begin{array}{l} \left[\begin{array}{c} u_{g}\\ u_{p}\\ e \end{array}\right]\sim \text{N}\left(\begin{array}{c} \left[\begin{array}{c} 0\\ 0\\ 0 \end{array}\right],\left[\begin{array}{ccc} G_{g} & 0 & 0\\ 0 & G_{p} & 0\\ 0 & 0 & R \end{array}\right] \end{array}\right) \end{array}$$

where the matrices ***G***_***g***_, ***G***_***p***_ and ***R*** are variance matrices for ***u***_***g***_ ***u***_***p***_ and ***e***, respectively. ***G***_***g***_ is known as the between environments variance/covariance matrix and is described in later sections. ***G***_***p***_ is assumed to be block diagonal given by $G_{p}={⊕}_{i=1}^{b}\sigma_{p_{i}}^{2}I_{q_{i}}$ where *b* is the number of components in ***u***_***p***_ and *q*_*i*_ is the number of effects in (length of) ***u***_***p***_***i***__. ***R*** is assumed to be block diagonal, so that $R={⊕}_{j=1}^{p}R_{j}$ where ***R***_*j*_ = var(***e***_*j*_) is the variance matrix for the residuals for the *j*^*th*^ environment. In the LMM of Smith et al. (2001b), spatial models are used for the residuals and the matrices ***R***_*j*_ correspond to separable autoregressive processes.

The random genetic effects ***u***_***g***_ comprise the variety effects nested within environments, and will be referred to as the VE effects. If we let *m* denote the total number of unique varieties across all environments, then the vector ***u***_***g***_ has length *mp*, which is ordered as varieties within environments. In this paper we allow for the use of pedigree information, so we partition the VE effects into additive and non-additive (residual VE) effects (Oakey et al., 2007) as follows:


$$\begin{array}{l} u_{g}=u_{a}+u_{e} \end{array}$$

It is assumed that var(***u***_***a***_) = ***G***_***a***_ ⊗ ***A*** where ***A*** is the numerator relationship matrix and ***G***_***a***_ is a *p* × *p* symmetric positive (semi)-definite matrix that will be referred to as the between environment additive genetic variance matrix. In terms of the non-additive effects, it is assumed that var(***u***_***e***_) = ***G***_***e***_ ⊗ ***I***_*m*_ where ***G***_***e***_ is a *p* × *p* symmetric positive (semi)-definite matrix that will be referred to as the between environment non-additive genetic variance matrix. The variance matrix of the total VE effects (that is, additive plus non-additive) is therefore given by:


(3) $$\begin{array}{l} \text{var}\left(u_{g}\right)=G_{g}=G_{a}⊗A+G_{e}⊗I_{m} \end{array}$$

Note that if no pedigree information is included in the analysis then ***u***_***g***_ = ***u***_***e***_ and ***G***_***g***_ = ***G***_***e***_ ⊗ ***I***_*m*_. Finally, the variance matrix for the data vector is given by:


(4) $$\begin{array}{l} \text{var}\left(y\right)=H=Z_{g}G_{g}{Z_{g}}^{⊤}+Z_{p}G_{p}{Z_{p}}^{⊤}+R \end{array}$$

The first step in fitting the model in Equation (1) is the estimation of the variance parameters associated with the random effects and residuals. We let **κ** denote the vector of (unknown) variance parameters and let *n*_κ_ be the associated number of parameters. We use residual maximum likelihood (REML) estimation which requires calculation of the REML scores for the elements of **κ**. These are given by:


(5) $$\begin{array}{l} U\left(\kappa_{i}\right)=-\frac{1}{2}\left[\text{tr}\left(P{\dot{H}}_{i}\right)-y^{⊤}P{\dot{H}}_{i}Py\right] \end{array}$$

where ***P*** = ***H***^−1^ − ***H***^−1^***X***(***X***^⊤^***H***^−1^***X***)^−^***X***^⊤^***H***^−1^ with (***X***^⊤^***H***^−1^***X***)^−^ being any generalised inverse of (***X***^⊤^***H***^−1^***X***). The “dot” notation indicates a derivative so that ${\dot{H}}_{i}=\partial H/\partial \kappa_{i},\;i=1\ldots n_{\kappa }$. The REML estimate of **κ** is obtained by equating the scores to zero and will be denoted by $\hat{\kappa }$. This typically requires an iterative scheme. A computationally efficient scheme is the average information algorithm of Gilmour et al. (1995) which is a Fisher scoring algorithm in which the average information matrix, $I_{A}$, is used instead of the expected information matrix, $I_{E}$. The elements of these matrices are given by:


(6) $$\begin{array}{l} I_{A}\left(\kappa_{i},\kappa_{j}\right)=\frac{1}{2}y^{⊤}P{\dot{H}}_{i}P{\dot{H}}_{j}Py\\ I_{E}\left(\kappa_{i},\kappa_{j}\right)=\frac{1}{2}\text{tr}\left(P{\dot{H}}_{i}P{\dot{H}}_{j}\right) \end{array}$$

Given the REML estimates of the variance parameters we can then compute empirical best linear unbiased estimates (EBLUEs) of the fixed effects and empirical best linear unbiased predictions (EBLUPs) of the random effects in Equation (1). In particular, the EBLUPs of the VE effects are given by ${\tilde{u}}_{g}=G_{g}{Z_{g}}^{⊤}Py$ and these have an associated prediction error variance of $\text{var}\left({\tilde{u}}_{g}-u_{g}\right)=G_{g}-G_{g}{Z_{g}}^{⊤}PZ_{g}G_{g}$. Note that in these equations the matrices ***G***_***g***_ and ***P*** are formed using the REML estimate $\hat{\kappa }$ of **κ**. We can then compute a model based reliability (Mrode and Thompson, 2005) for an individual VE effect prediction as the square of the correlation between the true effect and the EBLUP. For the *k*^*th*^ VE effect, this is obtained as:


(7) $$\begin{array}{l} \text{cor}{\left({ũ}_{g_{k}},u_{g_{k}}\right)}^{2}=1-\frac{{\left(G_{g}-G_{g}{Z_{g}}^{⊤}PZ_{g}G_{g}\right)}_{kk}}{{\left(G_{g}\right)}_{kk}} \end{array}$$

where the subscript “*kk*” indicates the *k*^*th*^ diagonal element of the associated matrix.

### 2.2. Information Based Diagnostic for Genetic Variance Parameter Estimation

An asymptotic variance matrix for the REML estimates of the variance parameters can be obtained as the inverse of the information matrix. This could either be the average information matrix or, more traditionally, the expected information matrix. For the purposes of developing a diagnostic, we use the latter as it does not depend on the data, the elements of which are given in Equation (6). In this paper the interest lies in the estimation of genetic variance parameters, so we partition the variance parameters as $\kappa ={\left({\kappa_{g}}^{⊤},{\kappa_{ḡ}}^{⊤}\right)}^{⊤}$, where **κ_*g*_** are the genetic variance parameters associated with ***G***_***a***_ and ***G***_***e***_ and **κ_ḡ_** are the remaining variance parameters, that is, associated with the peripheral random effects and residuals. We partition the full expected information matrix accordingly and write as:


(8) $$\begin{array}{l} I_{E}\left(\kappa ,\kappa^{⊤}\right)=\left[\begin{array}{cc} I_{E}\left(\kappa_{g},{\kappa_{g}}^{⊤}\right) & I_{E}\left(\kappa_{g},{\kappa_{ḡ}}^{⊤}\right)\\ I_{E}\left(\kappa_{ḡ},{\kappa_{g}}^{⊤}\right) & I_{E}\left(\kappa_{ḡ},{\kappa_{ḡ}}^{⊤}\right) \end{array}\right] \end{array}$$

The asymptotic variance matrix for ${\hat{\kappa }}_{g}$ can then be obtained as:


(9) $$\begin{array}{l} \Sigma \left(\kappa_{g},{\kappa_{g}}^{⊤}\right)={\left[I_{E}\left(\kappa_{g},{\kappa_{g}}^{⊤}\right)-I_{E}\left(\kappa_{g},{\kappa_{ḡ}}^{⊤}\right){\left(I_{E}\left(\kappa_{ḡ},{\kappa_{ḡ}}^{⊤}\right)\right)}^{-1}I_{E}\left(\kappa_{ḡ},{\kappa_{g}}^{⊤}\right)\right]}^{-1} \end{array}$$

Smith and Cullis (2018) recommend the use of factor analytic models for ***G***_***a***_ and ***G***_***e***_. Other possibilities include compound symmetric and unstructured forms. Irrespective of the form used, the parameters of interest are the variances and covariances in ***G***_***a***_ and ***G***_***e***_. The aim in this paper is to develop a diagnostic that reflects the information available to estimate these parameters but which does not require the fitting of the full LMM. In order to achieve this we apply some of the concepts from model-based design in which the aim is to search a design space for a configuration which is optimal in some sense under a pre-specified LMM. The latter includes specification of the terms in the model and also values for the variance parameters. Although the aim here is not to search a design space but rather to assess a particular design (dataset) we can proceed in a similar manner by considering a pre-specified LMM. In order to simplify computations but enable wide applicability we use a LMM that has a relatively simple structure for the non-genetic effects. In terms of the model in Equation (1) we assume that the fixed effects comprise a mean parameter for each environment (so that $\tau ={\left(\tau_{1},\tau_{2},\ldots ,\tau_{p}\right)}^{⊤}$) and we assume there are no peripheral effects so write:


(10) $$\begin{array}{l} y=X\tau +Z_{g}\left(u_{a}+u_{e}\right)+e \end{array}$$

where the design matrices are given by $X={⊕}_{j=1}^{p}1_{n_{j}}$ and $Z_{g}={⊕}_{j=1}^{p}{Z_{g}}_{j}$ where ***Z***_***g***_***j***__ is the *n*_*j*_ × *m* design matrix for the VE effects for environment *j* (= 1, …, *p*). The genetic variance matrices, ***G***_***a***_ and ***G***_***e***_, are assumed to have unstructured forms with *p*(*p* + 1)/2 unique variance parameters in each that are denoted by σ_*a*_*js*__ and σ_*e*_*js*__(*j* ≤ *s* = 1, …, *p*), respectively.

Finally, we assume that the residual variance matrices are given by $R_{j}=\sigma_{j}^{2}I_{n_{j}}$ so that $R={⊕}_{j=1}^{p}\sigma_{j}^{2}I_{n_{j}}$. The variance matrix for the data vector is then given by:


$$\begin{array}{l} H=Z_{g}\left(G_{a}⊗A+G_{e}⊗I_{m}\right){Z_{g}}^{⊤}+R \end{array}$$

and the unknown variance parameters are $\kappa ={\left({\kappa_{g}}^{⊤},{\kappa_{ḡ}}^{⊤}\right)}^{⊤}$ where **κ_*g*_** comprises σ_*a*_*js*__ and σ_*e*_*js*__ (*j* ≤ *s* = 1, …, *p*) and **κ_ḡ_** comprises $\sigma_{j}^{2}$ (*j* = 1, …, *p*). We then use pre-specified values of these parameters to compute the information matrix in Equation (8) and thence the variance matrix in Equation (9). The chosen variance parameters will be denoted **κ_*g*_0__** and **κ_ḡ_0__** and the resultant variance matrix denoted by $\Sigma \left({\kappa_{g}}_{0},{\kappa_{g}}_{0}^{⊤}\right)$. We then consider the D-optimality criterion of model-based design because it is used to search for designs that minimise the generalised variance of parameter estimates. In our setting we wish to measure the generalised variance of the genetic variance parameter estimates for a given dataset. This can be obtained for the complete set of genetic variance parameters as:


(11) $$\begin{array}{l} D=\frac{\log|\sum \left({\kappa_{g}}_{0},{\kappa_{g}}_{0}^{⊤}\right)|}{n_{\kappa_{g}}} \end{array}$$

where the vertical bar represents the determinant and *n*_κ_*g*__ is the number of genetic variance parameters which is used as a divisor to provide a scaling for comparisons across models and/or datasets.

Although the overall D-value is of interest, our focus is on individual environments and their relative contribution to the reliability of genetic variance parameter estimation. We therefore also compute a D-value for environment *j* (= 1, …, *p*) as:


(12) $$\begin{array}{l} D_{j}=\frac{\log|\sum \left({\kappa_{g}}_{0_{j}},{\kappa_{g}}_{0_{j}}^{⊤}\right)|}{n_{{\kappa_{g}}_{j}}} \end{array}$$

where $\Sigma \left({\kappa_{g}}_{0_{j}},{\kappa_{g}}_{0_{j}}^{⊤}\right)$ is the partition of $\Sigma \left({\kappa_{g}}_{0},{\kappa_{g}}_{0}^{⊤}\right)$ that relates to environment *j* and *n*_κ_*g*_*j*___ is the associated number of genetic variance parameters. In the case of models in which information on genetic relatedness is not used we have *n*_κ_*g*_*j*___ = *p*, and the parameters are the genetic variance for the environment and all *p* − 1 genetic covariances with other environments. In models in which the genetic effects are partitioned into additive and non-additive effects we have *n*_κ_*g*_*j*___ = 2*p*. To distinguish between these different genetic models we label the diagnostic values as $D_{j}$(A+I) if they correspond to a LMM with both additive (A) and non-additive (or independent, I) VE effects; $D_{j}$(I) if they correspond to the LMM with independent VE effects alone (that is, genetic relatedness is not used) or $D_{j}$(A) if they correspond to the LMM with additive VE effects alone. Irrespective of the genetic model used, the diagnostic values for all *p* environments can then be scrutinised in various ways in order to check for “problem” environments with large values, which represent those environments with large variance.

Finally, a further computational simplification can be made in the calculation of $\Sigma \left(\kappa_{g},{\kappa_{g}}^{⊤}\right)$ by using the marginal variance matrix for the genetic variance parameters rather than the conditional matrix as given in Equation (9). Thus, we can use:


(13) $$\begin{array}{l} \Sigma \left(\kappa_{g},{\kappa_{g}}^{⊤}\right)={\left[I_{E}\left(\kappa_{g},{\kappa_{g}}^{⊤}\right)\right]}^{-1} \end{array}$$

This is a reasonable simplification given that the non-genetic variance parameters in the pre-specified LMM are simply the residual variances so that the uncertainty associated with their estimation is likely to be small.

### 2.3. A Two-Stage Procedure

We first let *d*_*j*_ be the number of varieties in environment *j* (= 1, …, *p*) and define $d=\overset{p}{\underset{j=1}{\sum }}d_{j}$ to be the number of VE combinations present in the data. Then note that formation of $\Sigma \left({\kappa_{g}}_{0},{\kappa_{g}}_{0}^{⊤}\right)$ using Equations (9) or (13) involves calculating traces of matrices of dimension *n*. The dimensionality of the problem can be reduced by considering a two-stage approximation to the LMM as described in Gogel et al. (2018). Given the simple form for the model in Equation (10) and the associated variance matrices, we may expect little loss in using this approach and the benefit is a reduction in dimensionality from *n* (total number of plots in the dataset) to *d* (total number of VE combinations present).

In the first stage of the two-stage approach, a separate analysis is conducted for each environment in order to obtain predicted variety means and a measure of their uncertainty. In these analyzes the variety effects are regarded as fixed effects. The predicted means are combined across environments to form the data for the second stage analysis. We adopt the notation of Gogel et al. (2018) so let **η** denote the full *mp* × 1 vector of variety mean parameters for individual environments and let **η**_*d*_ be the *d* × 1 sub-vector corresponding to the VE combinations present in the data. Thus, we can write **η**_*d*_ = ***D*****η** where ***D*** is a *d* × *mp* indicator matrix that selects the appropriate elements. We let ${\hat{\eta }}_{d}$ be the vector of predicted variety means for individual environments from the first stage. In our setting the individual environment analyzes are particularly simple, involving only a single set of effects, namely the fixed variety effects, and the residual variance for environment *j* is simply $\sigma_{j}^{2}I_{n_{j}}$. This means that the variance matrix of ${\hat{\eta }}_{d}$ from the first stage is given by $\Omega ={⊕}_{j=1}^{p}\Omega_{j}$ where **Ω**_*j*_ is the *d*_*j*_ × *d*_*j*_ diagonal matrix given by $\sigma_{j}^{2}\text{diag}\left(1/r_{ji}\right)$, where *r*_*ji*_ is the number of plots of variety *i* in environment *j*.

The LMM for the second stage combined analysis of the *p* environments can then be written as:


(14) $$\begin{array}{l} y_{2}=X_{2}\tau +D\left(u_{a}+u_{e}\right)+\xi \end{array}$$

where $y_{2}={\hat{\eta }}_{d}$ from the first stage, and ***X***_**2**_ = ***D***(***I***_*p*_ ⊗ **1**_*m*_). In terms of the variance structures, var(***u***_***a***_) = ***G***_***a***_ ⊗ ***A*** and var(***u***_***e***_) = ***G***_***e***_ ⊗ ***I***_*m*_ (as in the one-stage analysis) and var(**ξ**) = **Ω** where this is known from the first stage. The variance matrix for the data vector in the second stage is then given by:


(15) $$\begin{array}{l} H_{2}=D\left(G_{a}⊗A+G_{e}⊗I_{m}\right)D^{⊤}+\Omega \end{array}$$

Elements of the expected information matrix for the variance parameters in the second stage LMM are then given by:


(16) $$\begin{array}{l} I_{2E}\left(\kappa_{i},\kappa_{j}\right)=\frac{1}{2}\text{tr}\left(P_{2}{\dot{H_{2}}}_{i}P_{2}{\dot{H_{2}}}_{j}\right) \end{array}$$

where $P_{2}={H_{2}}^{-1}-{H_{2}}^{-1}X_{2}{\left({X_{2}}^{⊤}{H_{2}}^{-1}X_{2}\right)}^{-1}{X_{2}}^{⊤}{H_{2}}^{-1}$. This now involves matrices of dimension *d* rather than *n*. As in the previous section 2.2 we do not actually conduct the two-stage analysis but compute the expected information matrix using Equation (16) for a given choice of variance parameter values. We then form the (marginal) variance matrix for the genetic variance parameter estimates using Equation (13) and denote the resultant matrix by $\Sigma_{2}\left({\kappa_{g}}_{0},{\kappa_{g}}_{0}^{⊤}\right)$. This is substituted into Equations (11) and (12) to compute the diagnostic.

A β-version of the R script file to calculate D-values is available upon request to the corresponding author.

## 3. Results

### 3.1. Application of Diagnostic to a MET Dataset

**Figure 1:**
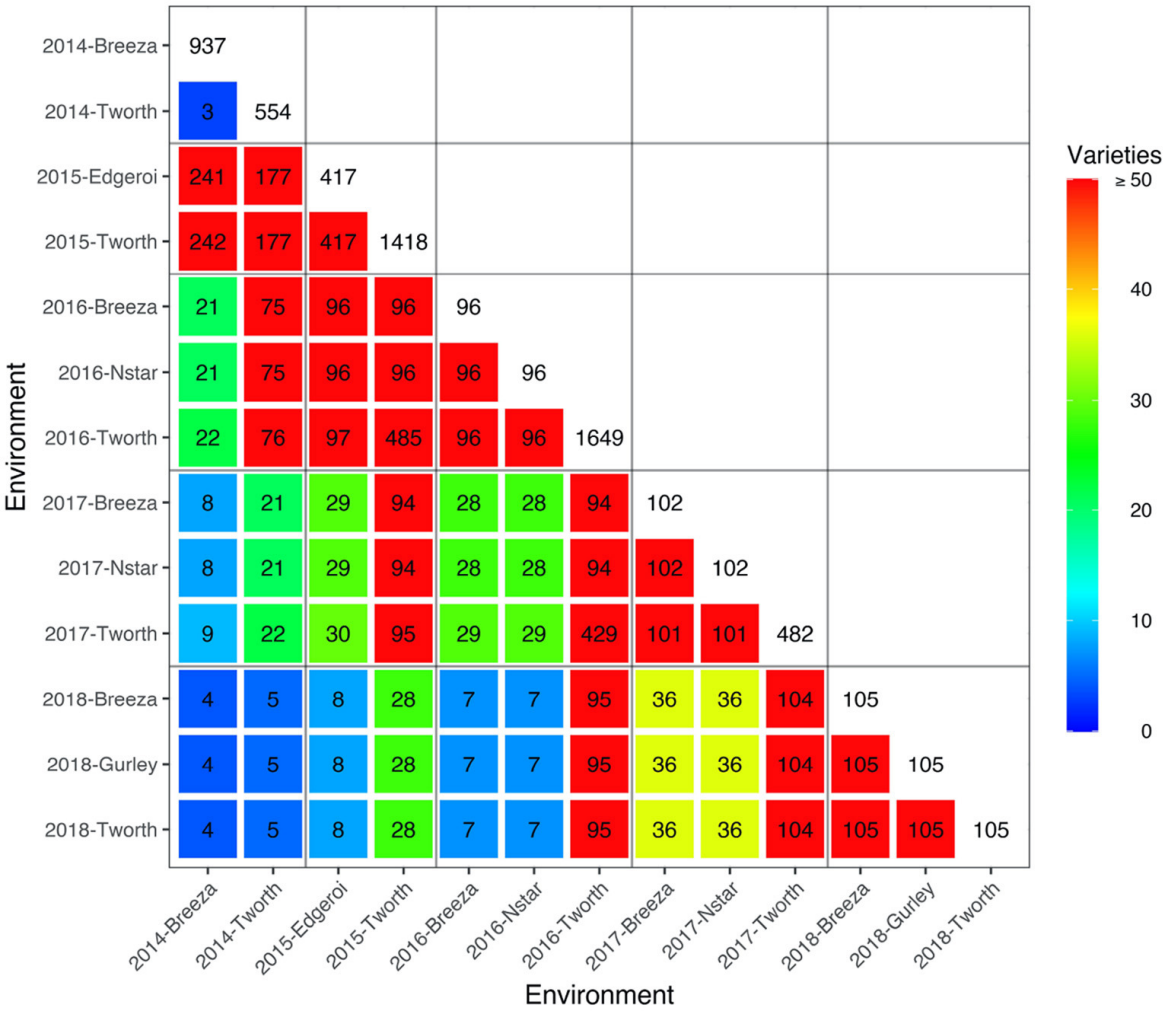
Durum example: Heatmap of the number of varieties in common between all pairs of environments in the durum MET dataset. The colors as referenced in the legends. The boxes along the diagonal show the number of unique varieties in individual environments. Boundaries for years are indicated by the grey horizontal and vertical lines.

The motivating MET dataset of this paper comes from the Durum Breeding Australia North program. Here we present some key summary information and apply the diagnostic procedure. Summary information for the durum wheat dataset is given in Table 1. The dataset comprised *m* = 3, 708 varieties from *n* = 9, 786 plots corresponding to 40 trials from breeding stages Stage 1 (S1) to Stage 3 (S3) across *p* = 13 environments sown between 2014 and 2018. The number of varieties per environment ranged from 96 to 1,649 with a median of 105. We note that there are *d* = 6, 168 variety by environment combinations present in the data, representing a nearly 40% reduction when using a 2-stage approach for computing the diagnostic. The pedigree information comprised 3,959 records and included all the varieties in the MET dataset. The inbreeding coefficients of the latter ranged from 0.750 to 0.998 with a mean of 0.911. The number of varieties in common between pairs of environments (displayed in a heatmap in Figure 1) ranged from 3 to 485 with a median of 36.

**Table 1:** Durum example: Summary of environments in the durum MET dataset.

**No**.	**Environment**	**Number of trials**				**Number of**	
		**S1**	**S2**	**S3**	**Total**	**Plots**	**Varieties**
1	2014-Breeza	3	0	0	3	1,296	937
2	2014-Tworth	2	0	0	2	700	554
3	2015-Edgeroi	0	4	0	4	864	417
4	2015-Tworth	6	4	0	10	2,052	1,418
5	2016-Breeza	0	0	1	1	192	96
6	2016-Nstar	0	0	1	1	192	96
7	2016-Tworth	6	3	1	10	2,448	1,649
8	2017-Breeza	0	0	1	1	204	102
9	2017-Nstar	0	0	1	1	204	102
10	2017-Tworth	0	3	1	4	1,004	482
11	2018-Breeza	0	0	1	1	210	105
12	2018-Gurley	0	0	1	1	210	105
13	2018-Tworth	0	0	1	1	210	105
Total		17	14	9	40	9,786	3,708

*Number of: trials for each stage of testing (S1, S2, and S3), total trials, plots and varieties*.

Given that the analysis of the durum data for the purposes of variety selection would proceed using a LMM with the partitioning of the VE effects into additive and non-additive effects, we computed the $I_{2E}$ based on this model (so that *n*_κ_*g*__ = 182). The values of the variance parameters for calculation of the diagnostic were set to:


$$\begin{array}{l} \sigma_{a_{js}}=0.10;\;j=s=1,\ldots ,p\\ \;=0.08;\;j<s=1,\ldots ,p\\ \sigma_{e_{js}}=0.05;\;j=s=1,\ldots ,p\\ \;=0.04;\;j<s=1,\ldots ,p\\ \sigma_{j}^{2}=0.15;\;j=1,\ldots ,p \end{array}$$

These values were chosen as being both representative of actual estimates from historical analyzes that are often encountered in practise and of a magnitude that could allow the diagnostic to provide good discrimination between environments. In particular, we have set the additive genetic variance to 80% of the total genetic variance (see Equation 3) for each environment and therefore 20% for the non-additive genetic variance), and a between environments correlation of 0.8 for both additive and non-additive VE effects.

The $D_{j}$(A+I)-values for each environment from this pre-specified LMM are given in Table 2. The environments 2016-Tworth and 2015-Tworth had the smallest $D_{j}$(A+I)-values and therefore the greatest information to estimate genetic variance parameters. Whereas, 2018-Tworth, 2018-Gurley, and 2018-Breeza had the largest $D_{j}$(A+I)-values and therefore the least information to estimate genetic variance parameters.

**Table 2:** Durum example: Diagnostic $D_{j}$-values based on LMMs with additive and non-additive VE effects [$D_{j}$(A+I)] and those based on LMMs with additive VE effects alone [$D_{j}$(A)].

**Environment**	**High**		**Low**	
	$D_{j}$ **(A+I)**	$D_{j}$ **(A)**	$D_{j}$ **(A+I)**	$D_{j}$ **(A)**
2016-Tworth	–8.28	–9.25	–7.82	–8.60
2015-Tworth	–8.27	–9.27	–7.82	–8.61
2017-Tworth	–8.03	–9.04	–7.45	–8.25
2015-Edgeroi	–7.95	–8.94	–7.30	–8.09
2017-Breeza	–7.65	–8.69	–6.95	—7.80
2017-Nstar	–7.65	–8.69	–6.95	–7.80
2014-Breeza	–7.56	–8.61	–6.74	–7.47
2016-Breeza	–7.51	–8.53	–6.76	–7.61
2016-Nstar	–7.51	–8.53	–6.76	–7.61
2014-Tworth	–7.43	–8.51	–6.72	–7.53
2018-Breeza	–7.39	–8.55	–6.67	–7.59
2018-Gurley	–7.39	–8.55	–6.67	–7.59
2018-Tworth	–7.39	–8.55	–6.67	–7.59

*High and low parameter values of additive variance (80 and 40%, respectively) and between environments genetic correlation (0.8 and 0.4, respectively) are used. Environments are ordered in ascending order on their $D_{j}$(A+I)-values (High)*.

**Figure 2:**
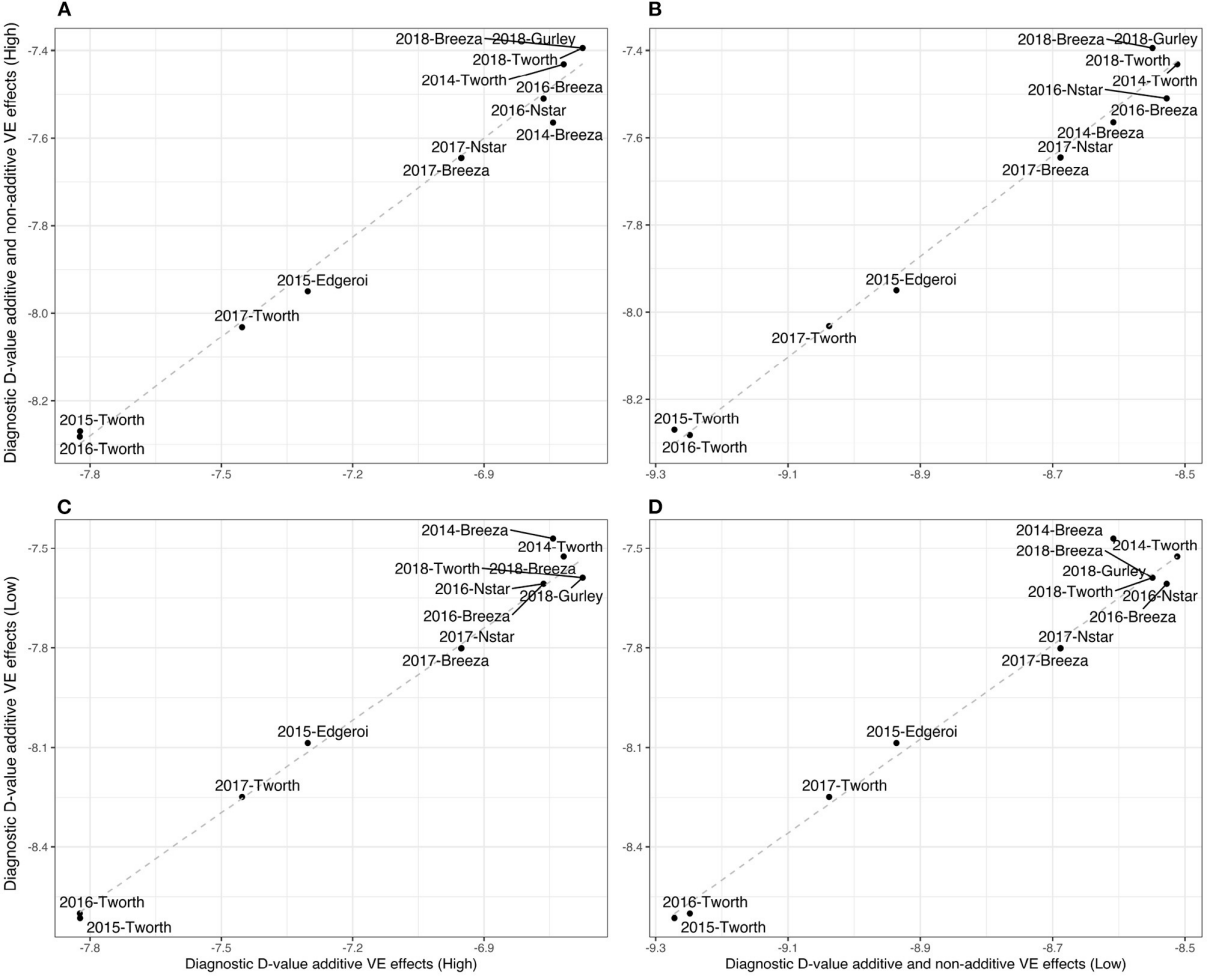
Durum example: Comparisons of Diagnostic $D_{j}$-values based on LMMs with additive and non-additive VE effects [$D_{j}$(A+I)] and those based on LMMs with additive VE effects alone ($D_{j}$(A+I)) for high and low parameter values of additive variance (80 and 40%, respectively) and between environments genetic correlation (0.8 and 0.4, respectively). **(A)** $D_{j}$(A+I) against $D_{j}$(A) values using the high parameter values, **(B)** $D_{j}$(A+I) against $D_{j}$(A+I) values using high and low parameter values, respectively, **(C)** $D_{j}$(A) against $D_{j}$(A) using high and low parameter values, respectively, and **(D)** $D_{j}$(A) against $D_{j}$(A+I) using low parameter values.

Given the high percentage of additive genetic variance we also computed the simpler diagnostic, based on a LMM with additive VE effects alone. For this diagnostic *n*_κ_*g*__ = 91 representing a four-fold reduction in the number of elements in $I_{2E}\left(\kappa_{i},\kappa_{j}\right)$ and hence a significant savings in computation. The resultant $D_{j}$(A)-values are presented in Figure 2A and Table 2, which show little difference compared with the $D_{j}$(A+I)-values. In particular, Figure 2A shows an almost 1:1 relationship.

To investigate the robustness of the diagnostic we also inspect the $D_{j}$(A+I)- and $D_{j}$(A)-values with parameters set to those which are at the lower end of those seen in practise. The values of the variance parameters for calculation of the diagnostic were set to:


$$\begin{array}{l} \sigma_{a_{js}}=0.05;\;j=s=1,\ldots ,p\\ \;=0.02;\;j<s=1,\ldots ,p\\ \sigma_{e_{js}}=0.15;\;j=s=1,\ldots ,p\\ \;=0.06;\;j<s=1,\ldots ,p\\ \sigma_{j}^{2}=0.15;\;j=1,\ldots ,p \end{array}$$

In particular, we have set the additive genetic variance to 40% of the total genetic variance (see Equation 3) for each environment and therefore 60% for the non-additive genetic variance, and a between environments correlation of 0.4 for both additive and non-additive VE effects. The resultant $D_{j}$-values are presented in both Figure 2 and Table 2. Once again there is little difference in the rankings of environments for $D_{j}$(A+I) compared with $D_{j}$(A) (Figure 2D). Additionally the rankings were robust to the two choices of variance parameters (high and low) used in forming the diagnostic, with the only noticeable change being associated with 2014-Breeza (Figures 2B,C).

**Figure 3:**
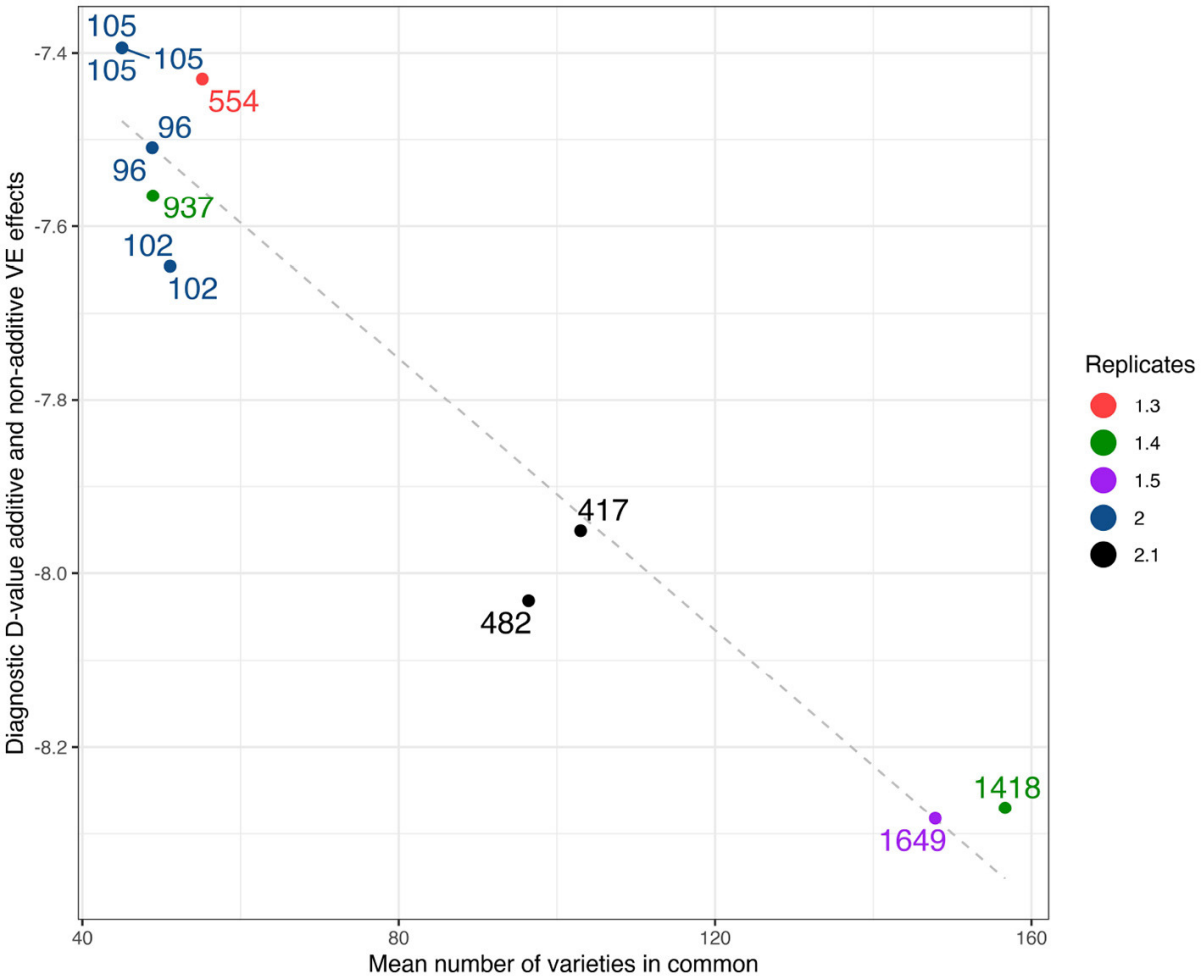
Durum example: Diagnostic $D_{j}$(A+I)-values based on LMM with additive and non-additive VE effects plotted against mean number of varieties in common. Labels show the total number of varieties, colors as represented in legend show the mean number of replicates.

As a comparison with the historical measure of variety connectivity the $D_{j}$(A+I) values computed using the high set of parameters have been plotted against mean connectivity in Figure 3. Two other structural characteristics of the environments are indicated on this plot, namely the number of varieties grown and the mean replication per variety. The figure shows that the diagnostic values encompass numerous structural elements of the environments.

### 3.2. Simulation Studies to Investigate the Performance of the Diagnostic

#### 3.2.1. LMM Without Pedigree Information

Within the framework of the LMM with independent VE effects it was previously thought that variety connectivity was a key driver of the reliability of variance parameter estimation and that this in turn affected the reliability of predictions of VE effects. We therefore consider a simulation study in which a range of connectivity levels is examined and assess the performance of both variety connectivity and the D-optimality diagnostic. For simplicity, and without loss of generality, we use *p* = 2 environments and label these as Env1 and Env2. Each environment has the same number of varieties (so that *d*_1_ = *d*_2_), and we vary the number of varieties in common (which is given by *c* = *d* − *m*). We assume the trials in Env1 and Env2 comprise 3 replicates and consider 4 sizes (Tsize) of trial corresponding to different numbers of varieties, namely *d*_1_(= *d*_2_)= 12, 24, 48 and 96 so that *n*_1_(= *n*_2_)= 36, 72, 144 and 288.

The simulation study for the first trial size (Tsize=12) is described in the following. We consider the connectivity levels *c* = 2, 4…12 (increments of 2). The maximum total number of varieties across Env1 and Env2 is *m* = 22, corresponding to *c* = 2. We label these varieties as V1 - V22. We assume that the 12 varieties in Env1 are always V1 - V12. The 12 varieties in Env2 are then V1 - V12 for *c* = 12; V2 - V13 for *c* = 11 and so on to V11 - V22 for *c* = 2. Our focus is on Env1 because this contains the same varieties across all connectivity levels so allows a fair comparison across these levels. The underlying LMM is as in Equation (10) but with independent VE effects alone (that is, without the additive VE effects) so that *n*_κ_*g*__ = 3. Given the data structure for each value of *c* and some pre-specified variance parameters, we can compute the diagnostic for Env1 which will be denoted $D_{1c}$(I). For the purposes of both the calculation of $D_{1c}$(I) and of data generation in the simulation study we chose the values of the variance parameters to be ${\kappa_{g}}_{0}={\left(\sigma_{e_{11_{0}}}=0.2,\sigma_{e_{12_{0}}}=0.16,\sigma_{e_{22_{0}}}=0.2\right)}^{⊤}$ and ${\kappa_{ḡ}}_{0}={\left(\sigma_{1_{0}}^{2}=0.15,\sigma_{2_{0}}^{2}=0.15\right)}^{⊤}$. The diagnostic is calculated using the two-stage formula for expected information, that is, as in Equation (16) and is given by:


(17) $$\begin{array}{l} D_{1c}\text{(I)}=\frac{\log|\Sigma_{c}\left(\left(\sigma_{e_{11_{0}}},\sigma_{e_{12_{0}}}\right),{\left(\sigma_{e_{11_{0}}},\sigma_{e_{12_{0}}}\right)}^{⊤}\right)|}{2} \end{array}$$

In the simulation study, the steps for the *t*^*th*^ simulation (*t* = 1…*N*) are as follows:

1. Generate the random genetic effects ***u***_***e***_ and residuals ***e*** as per the LMM in Equation (10) and for the pre-specified variance parameters **_κ_*g*0__** and **_κḡ_0__**. In terms of the fixed effects, without loss of generality we choose τ_1_ = τ_2_ = 0. Note that we generate 2*m* genetic effects, where *m* = 22 which corresponds to the maximum total number of varieties across all connectivity levels. We denote the resultant vector for simulation *t* by ***u***_*e*_*t*__. The residuals for simulation *t* are denoted by ***e***_*t*_ which is a vector of length *n*_1_ + *n*_2_ = 72.
2. For the connectivity level *c*, we subset the appropriate 24 elements of ***u***_*e*_*t*__. We will label the associated vector as ***u***_*e*_*tc*__. We then form the data vector and fit the LMM as in Equation (10), without the inclusion of pedigree information. We save the REML estimates of the genetic variance parameters, denoting these as ${\hat{\sigma }}_{e_{11_{tc}}},{\hat{\sigma }}_{e_{12_{tc}}},{\hat{\sigma }}_{e_{22_{tc}}}$ and save the EBLUPs of the genetic effects, denoting these as ${\tilde{u}}_{e_{tc}}$.
3. Repeat step 2. for each value of *c*

A total of *N* = 2, 000 simulations was conducted for each trial size by variety connectivity combination. The simulation based diagnostics and reliabilities were only computed for the LMMs in Step 2. that achieved convergence (with one update if required) and resulted in a positive definite form for the REML estimate of ***G***_***e***_. All models in this paper were fitted using the ASReml-R package (Butler et al., 2017) within R (R Core Team, 2020).

The simulations were conducted in order to obtain two main quantities of interest for each value of *c*, namely a measure of the reliability of the genetic variance parameter estimates and a measure of the reliability of the predicted variety effects for Env1. For the former, we computed a simulation based equivalent of the diagnostic in Equation (17), namely:


(18) $$\begin{array}{l} D_{1c}^{S}\text{(I)}=\frac{\log|V_{c}\left(\left({\hat{\sigma }}_{e_{11_{c}}},{\hat{\sigma }}_{e_{12_{c}}}\right),{\left({\hat{\sigma }}_{e_{11_{c}}},{\hat{\sigma }}_{e_{12_{c}}}\right)}^{⊤}\right)|}{2} \end{array}$$

where the determinant is with respect to the sample variance/covariance matrix of the REML estimates of the genetic variance parameters for Env1. In terms of the variety predictions, we computed the reliability of the EBLUPs for the 12 varieties that were always present in Env1, namely V1 - V12. For each value of *c*, the reliability for variety *k*(= 1…12) in Env1 was computed as the square of the sample correlation between the true (generated) effects (element of ***u***_*e*_*tc*__ for the variety and Env1) and the EBLUPs (element of ${\tilde{u}}_{e_{tc}}$ for the variety and Env1). This will be denoted $R_{kc}^{S}$.

Noting that the simulation based reliabilities ($R_{kc}^{S}$) of the variety predictions take into account the uncertainty in having to estimate the variance parameters, we compute analogues values that assume known variance parameters. These reliabilities therefore reflect the maximum possible values against which we can measure the loss attributable to variance parameter estimation. This was achieved by fitting, for each value of *c*, the LMM as per Equation (10) but with the variance parameters fixed at the value ***κ***_0_. We then computed the model based reliability for variety *k*(= 1…12) in Env1 using Equation (7) but because this has been computed with respect to known variance parameters (not REML estimates) we will call it the design based reliability ($R_{kc}^{D}$). We calculated the associated loss for the EBLUP reliabilities as:


(19) $$\begin{array}{l} R_{kc}^{D}-R_{kc}^{S} \end{array}$$

Finally we summarise these by taking means across the varieties in Env1 that were also present in Env2. We restrict the results to this set of varieties because in any MET analysis, there is a fundamental difference between varieties that were present in multiple environments (so-called connected varieties) and those that were present in a single environment only. The MET analysis, compared with separate analyzes of individual environments, has the potential to improve the reliability of predictions for connected varieties through the use of additional data. This is not the case for varieties present in a single environment only. Hence our focus is the connected varieties.

#### 3.2.2. Results of Simulation Without Pedigree Information

**Figure 4:**
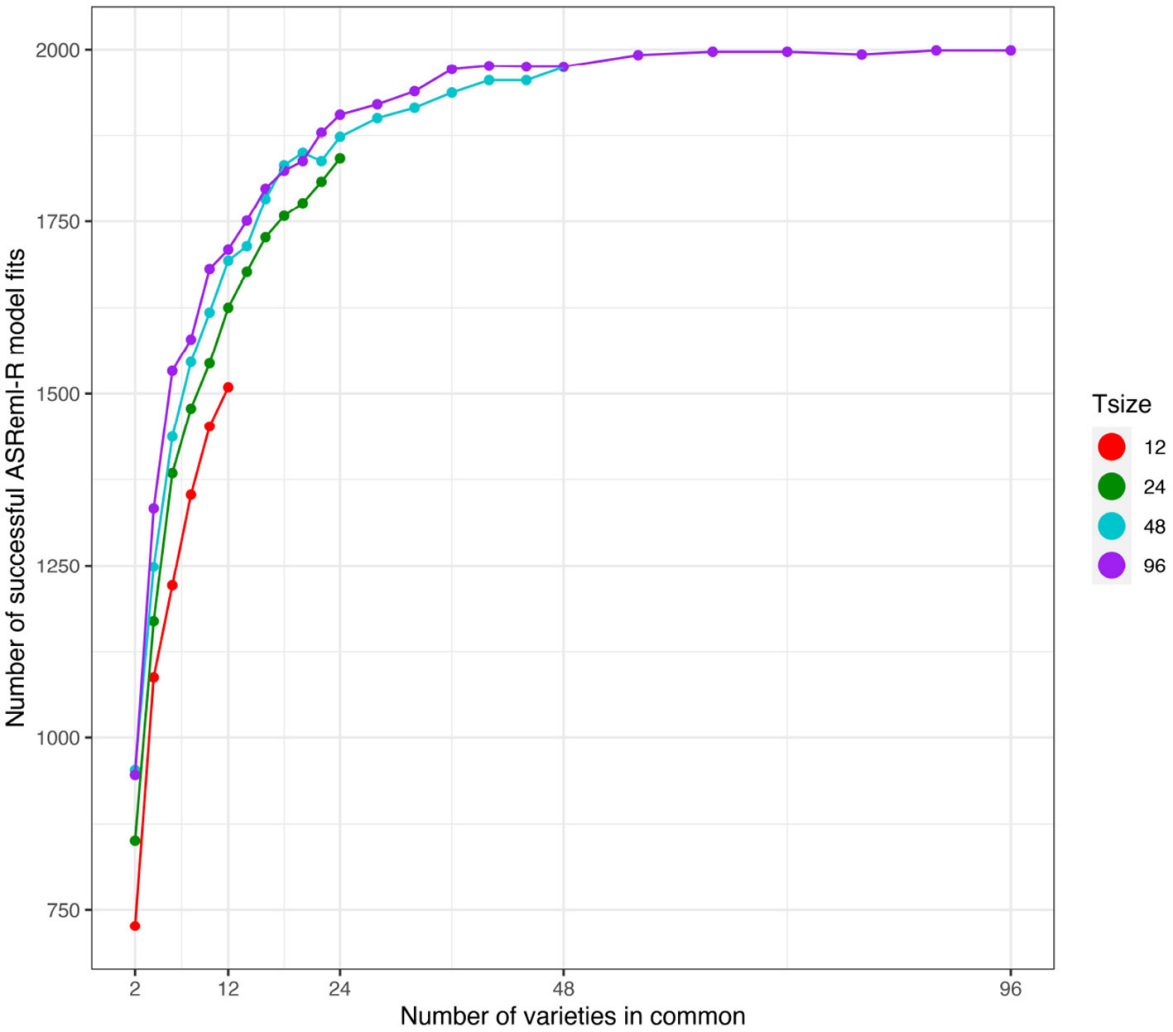
Independent VE effects simulation study: Number of successful model fits from *N* = 2, 000 simulations plotted against number of varieties in common for four trial sizes (trials with 12, 24, 48, and 96 varieties). Trial sizes (Tsize) are represented using different colours. Each point within Tsize corresponds to a different level of variety connectivity which ranges from *c* = 2 up to the number of varieties in a trial (representing 100% connectivity between the two trials).

First we note that the number of simulations in which the model fitting was successful (as defined in section 3.2.1) was strongly related to the number of varieties in common between the two environments (see Figure 4). The number of successful model fits for the connectivity level of *c* = 2 was particularly low and additionally the results were found to be unreliable. Therefore, in what follows, the results for this connectivity level have been excluded.

**Figure 5:**
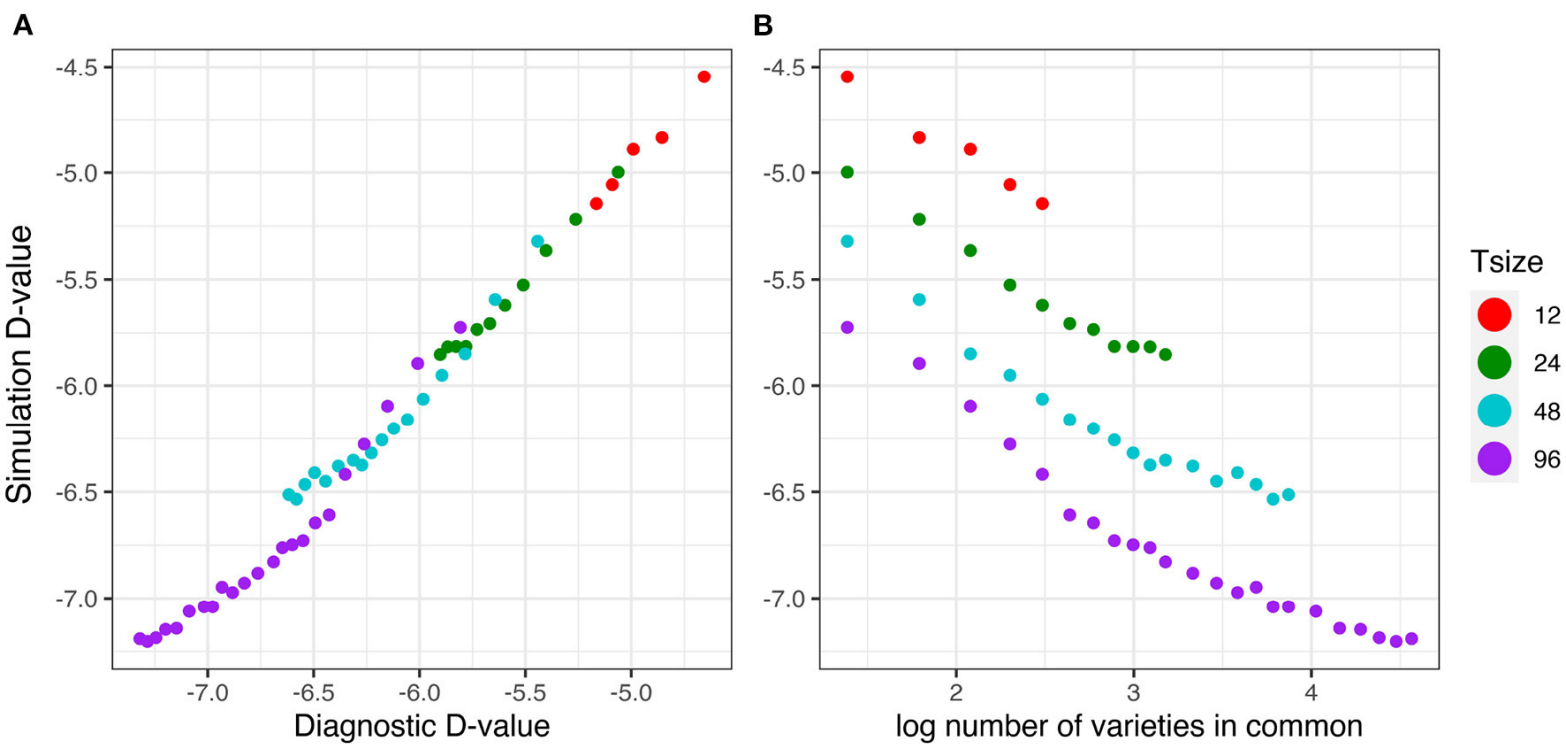
Independent VE effects simulation study: Simulation based $D_{1c}^{S}$(I)-values plotted against **(A)** diagnostic $D_{1c}$(I)-values and **(B)** log number of varieties in common for four trial sizes (trials with 12, 24, 48, and 96 varieties) and a sequence of connectivity levels. Trial sizes (Tsize) are represented using different colours. Each point within Tsize corresponds to a different level of variety connectivity which ranges from *c* = 4 up to the number of varieties in a trial (representing 100% connectivity between the two trials).

The relationship between the diagnostic $D_{1c}$(I)-values (from Equation 17) and the simulation based equivalent $D_{1c}^{S}$(I)-values (from Equation 18) is shown in Figure 5A. This good agreement shown in Figure 5A clearly indicates that the diagnostic performs well in terms of forecasting the level of uncertainty in genetic variance parameter estimation. Figure 5B shows that there is a decreasing linear relationship between (log) variety connectivity and the uncertainty in genetic variance parameter estimation, but this is only within a given trial size, that is, for a given number of varieties. The connectivity measure fails for comparisons involving trials with different number of varieties.

**Figure 6:**
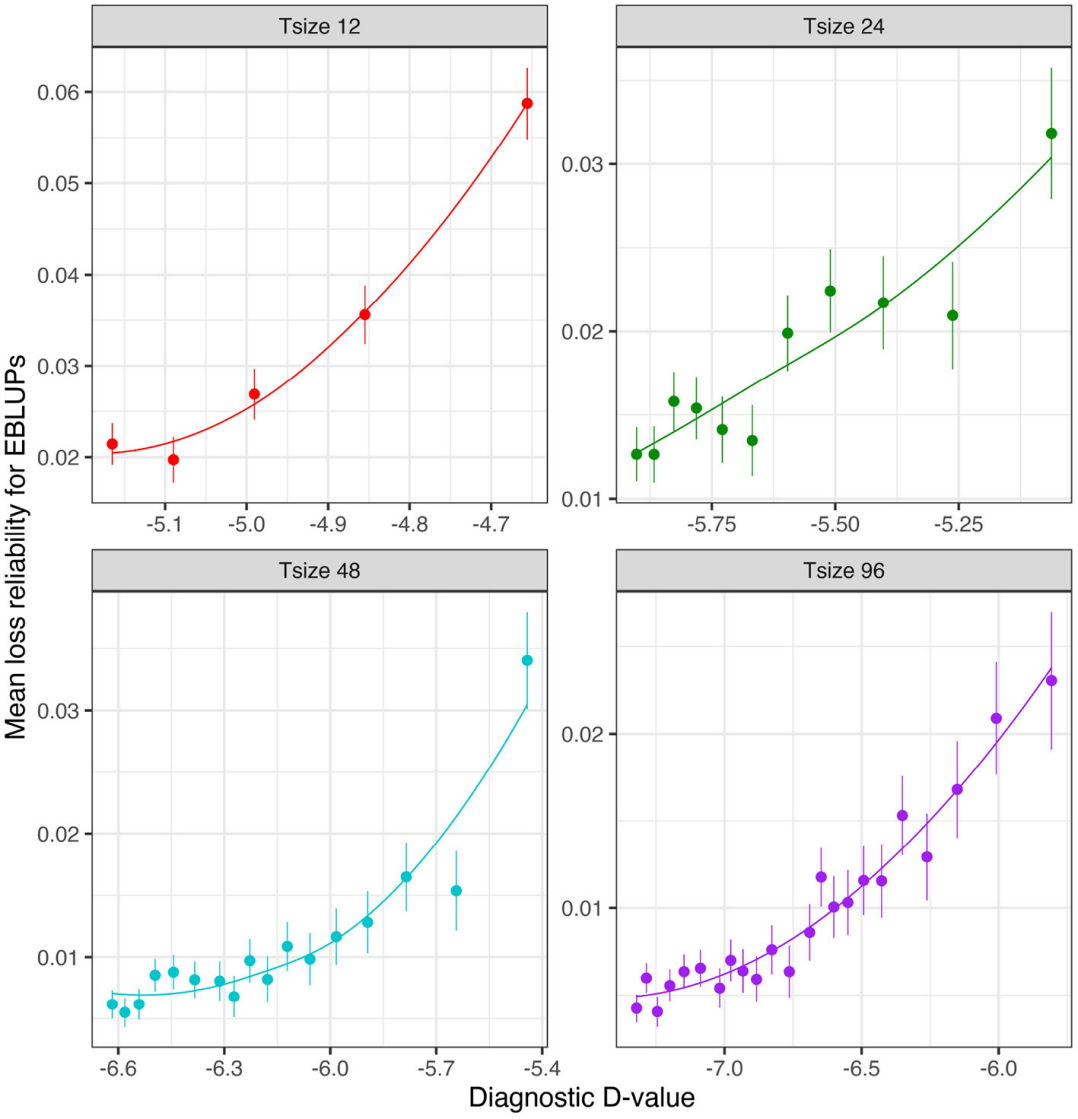
Independent VE effects simulation study: Mean loss in reliability of the EBLUPs of VE effects for Env1 for those varieties that were present in both environments. Each panel corresponds to a different trial size (trials with 12, 24, 48, and 96 varieties) and the points correspond to a sequence of connectivity levels. Also shown are standard errors for each mean (vertical lines) and a loess smoother through the means for each Tsize.

**Figure 7:**
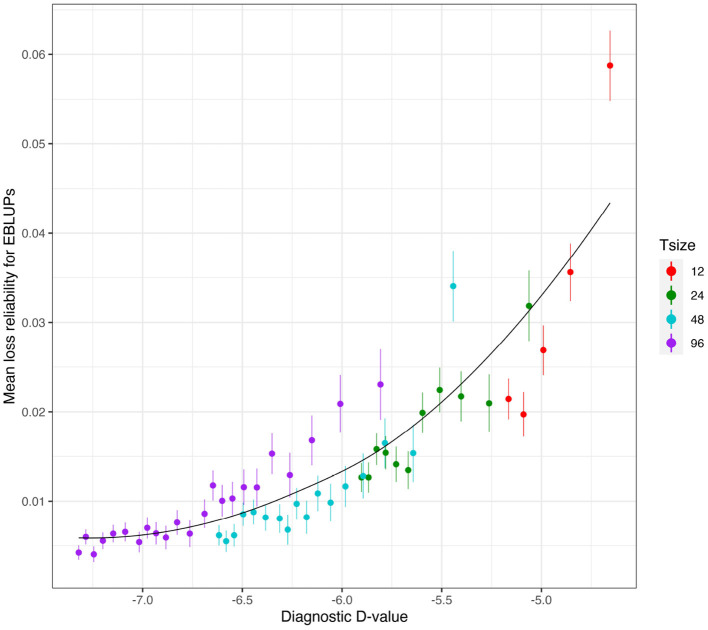
Independent VE effects simulation study: Mean loss in reliability of the EBLUPs of VE effects for Env1 for those varieties that were present in both environments. The colours correspond to different trial sizes (trials with 12, 24, 48, and 96 varieties) and the points for each colour correspond to a sequence of connectivity levels. Also shown are standard errors for each mean (vertical lines) and a loess smoother through all the means.

Figure 6 shows the mean losses in reliability of the EBLUPs of VE effects for Env1 for those varieties that were present in both environments. These are plotted against the diagnostic $D_{1c}$(I)-values, with a separate panel for each trial size. Each point has been supplemented with a standard error of the mean (SEM) which was based on a pooled estimate of error across all trial sizes and connectivity levels. Thus differences in SEM reflect differences in the numbers of varieties used to compute the means (that is, differences in connectivity). The panels in this figure show that, for a given trial size, the loss in reliability of EBLUPs is well predicted by the diagnostic $D_{1c}$(I)-values. This also holds across trial sizes, although the relationship is more variable (Figure 7).

Results displayed in Figures 4, 5, 7 have been extracted for the “best case” scenario of 100% connectivity for each Tsize and are presented in Table 3. This again shows the good agreement between the diagnostic $D_{1c}$(I)-values and the simulation based $D_{1c}^{S}$(I)-values and the relationship between the diagnostic and the loss in reliability of VE predictions. It also shows that, even with 100% connectivity, there were substantial problems with the smallest trial size in terms of all criteria (number of successful model fits, reliability of genetic variance parameter estimates and reliability of VE effect predictions).

**Table 3:** Independent VE effects simulation study: Summary of key results for each trial size and the case of 100% connectivity between the two trials: Simulation based $D_{1c}^{S}$(I)-values; diagnostic $D_{1c}$(I)-values; mean loss in reliability of EBLUPs of VE effects for Env1 (with associated standard error); number of successful model fits out of *N* = 2, 000 simulations.

**Tsize**	**Varieties in**	$D_{1c}^{S}$ **(I)**	$D_{1c}$ **(I)**	**EBLUP reliability**		**Successful**
	**common**					**model fits**
				**loss**	**se**	
12	12	–5.15	–5.16	0.021	0.0023	1,509
24	24	–5.85	–5.90	0.013	0.0016	1,842
48	48	–6.51	–6.62	0.006	0.0011	1,974
96	96	–7.19	–7.32	0.004	0.0008	1,999

#### 3.2.3. LMM With Pedigree Information

We then extended the simulation study in order to assess the performance of the diagnostic in terms of correlated VE effects. To simplify the simulations, and without loss of generality, we considered the LMM as in Equation (1) but without the non-additive VE effects, so that as in section 3.2.1, *n*_κ_*g*__ = 3. The set-up for the study is the same as in section 3.2.1 but we only consider the two larger trial sizes, namely *d*_1_(= *d*_2_)= 48 and 96 so that *n*_1_(= *n*_2_)= 144 and 288. Across the range of connectivity levels the total number of varieties required for the simulation was 191 (corresponding to *c* = 2 for the trial size of 96) and we label these as V1-V191. The simulation study requires a numerator relationship matrix (***A***) for these varieties. We therefore chose V1-V191 from the actual lines in Stage 3 (S3) and Stage 4 (S4) in 2018 and 2017 in the durum data and computed ***A*** from the associated pedigree information. For the chosen subset of varieties, the inbreeding coefficient ranged from 0.938 to 0.969 with a mean of 0.957.

For the purposes of both the calculation of $D_{1c}$(A) and of data generation in the simulation study we chose the values of the variance parameters to be ${\kappa_{g}}_{0}={\left(\sigma_{a_{11_{0}}}=0.1,\sigma_{a_{12_{0}}}=0.08,\sigma_{a_{22_{0}}}=0.1\right)}^{⊤}$ and ${\kappa_{ḡ}}_{0}={\left(\sigma_{1_{0}}^{2}=0.15,\sigma_{2_{0}}^{2}=0.15\right)}^{⊤}$.

#### 3.2.4. Results of Simulation With Pedigree Information

**Figure 8:**
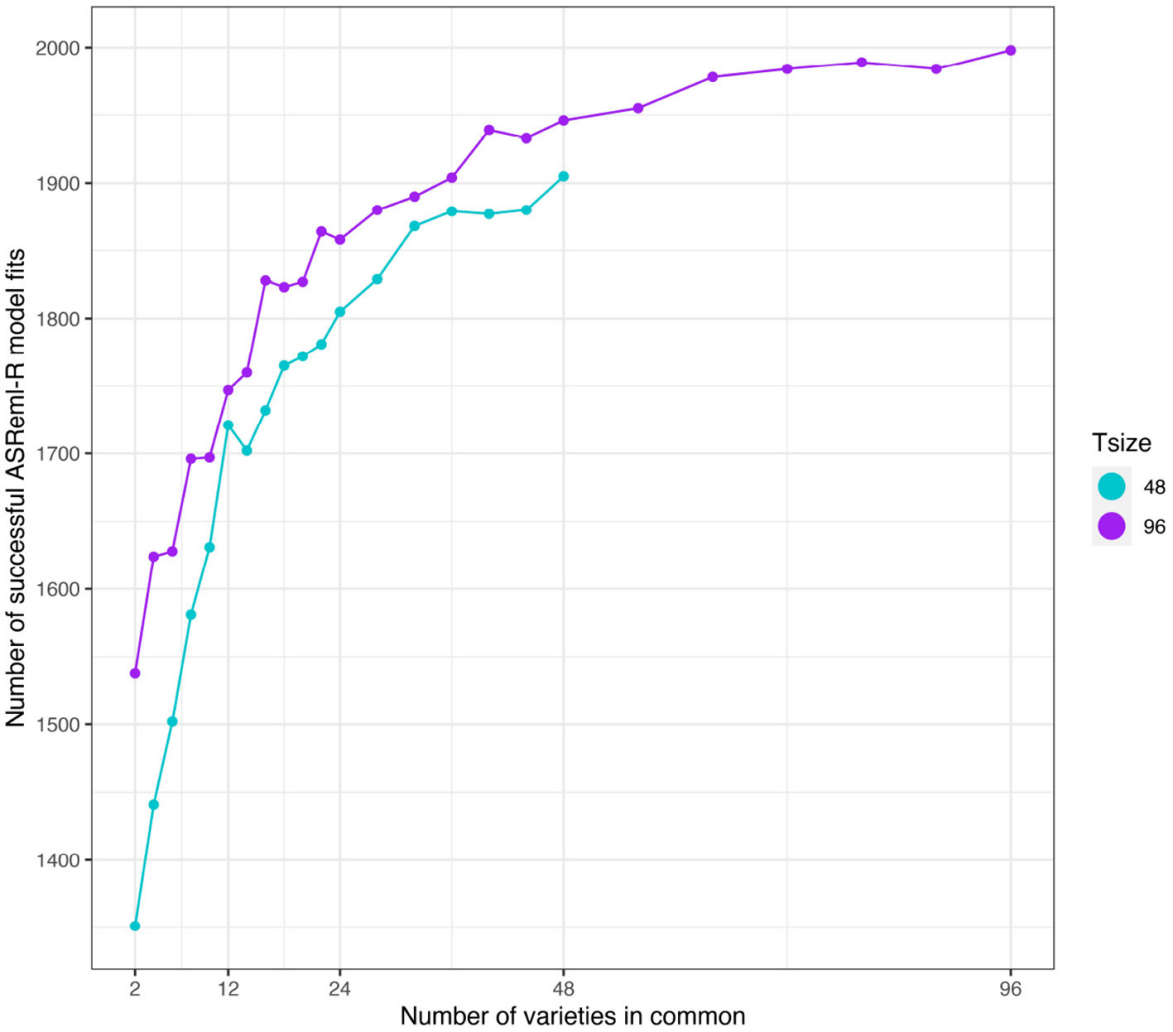
Additive VE effects simulation study: Number of successful model fits from *N* = 2, 000 simulations plotted against number of varieties in common for two trial sizes (trials with 48 and 96 varieties). Trial sizes (Tsize) are represented using different colours. Each point within Tsize corresponds to a different level of variety connectivity which ranges from *c* = 2 up to the number of varieties in a trial (representing 100% connectivity between the two trials).

As in the independent VE effects study, the number of simulations in which the model fitting was successful was related to the number of varieties in common between the two environments (see Figure 8). However, a key difference was that the number of successful model fits for the connectivity level of *c* = 2 was reasonable so these results have been included in what follows.

**Figure 9:**
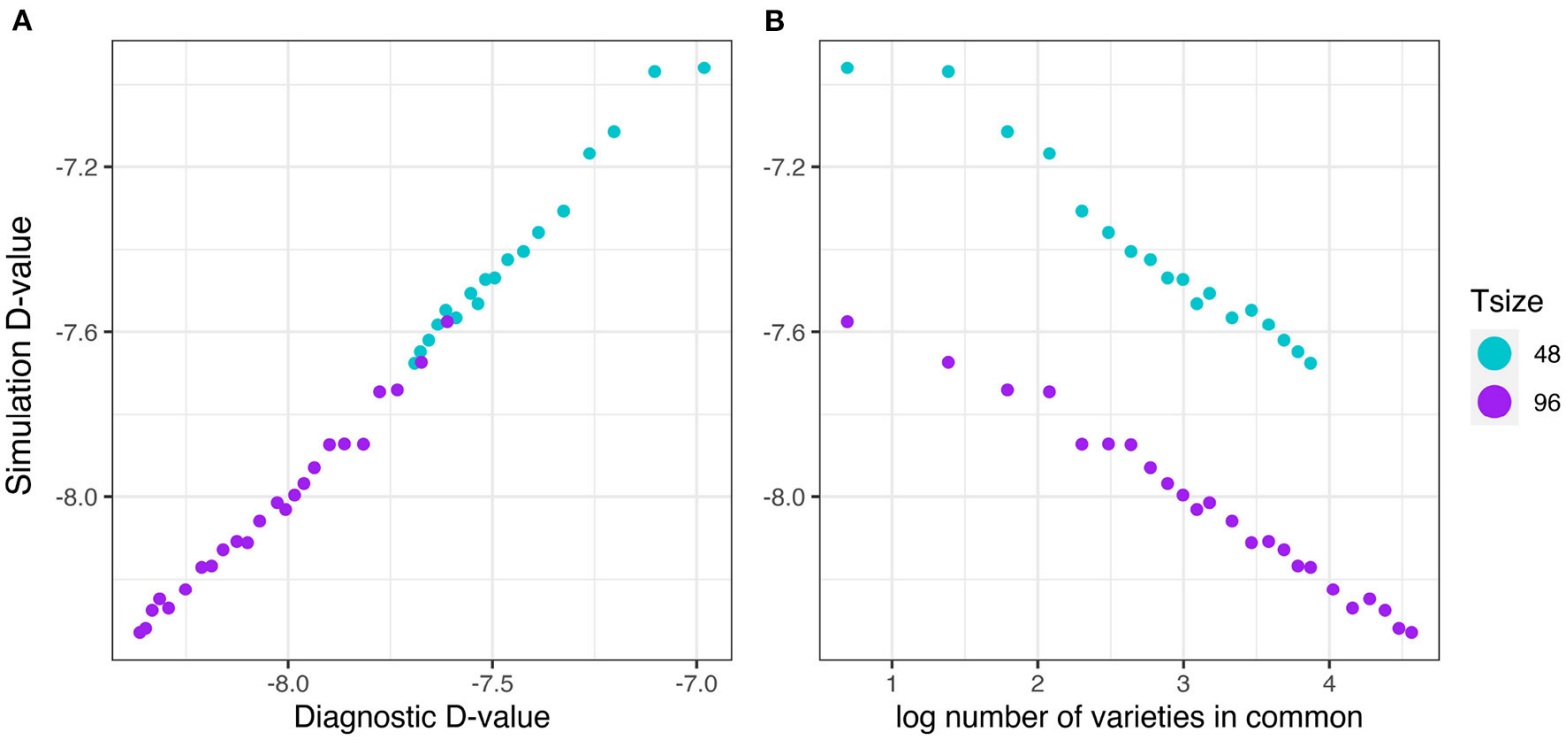
Additive VE effects simulation study: Simulation based $D_{1c}^{S}$(A)-values plotted against **(A)** diagnostic $D_{1c}$(A)-values and **(B)** log number of varieties in common for two trial sizes (trials with 48 and 96 varieties) and a sequence of connectivity levels. Trial sizes (Tsize) are represented using different colours. Each point within Tsize corresponds to a different level of variety connectivity which ranges from *c* = 2 up to the number of varieties in a trial (representing 100% connectivity between the two trials).

The results are presented in the same format as in section 3.2.1. The good agreement shown in Figure 9A clearly indicates that the diagnostic performs well in terms of forecasting the level of uncertainty in genetic variance parameter estimation in the presence of pedigree information. Figure 9B shows that there is a decreasing linear relationship between (log) variety connectivity and the uncertainty in genetic variance parameter estimation, but this only holds for trials with the same number of varieties.

**Figure 10:**
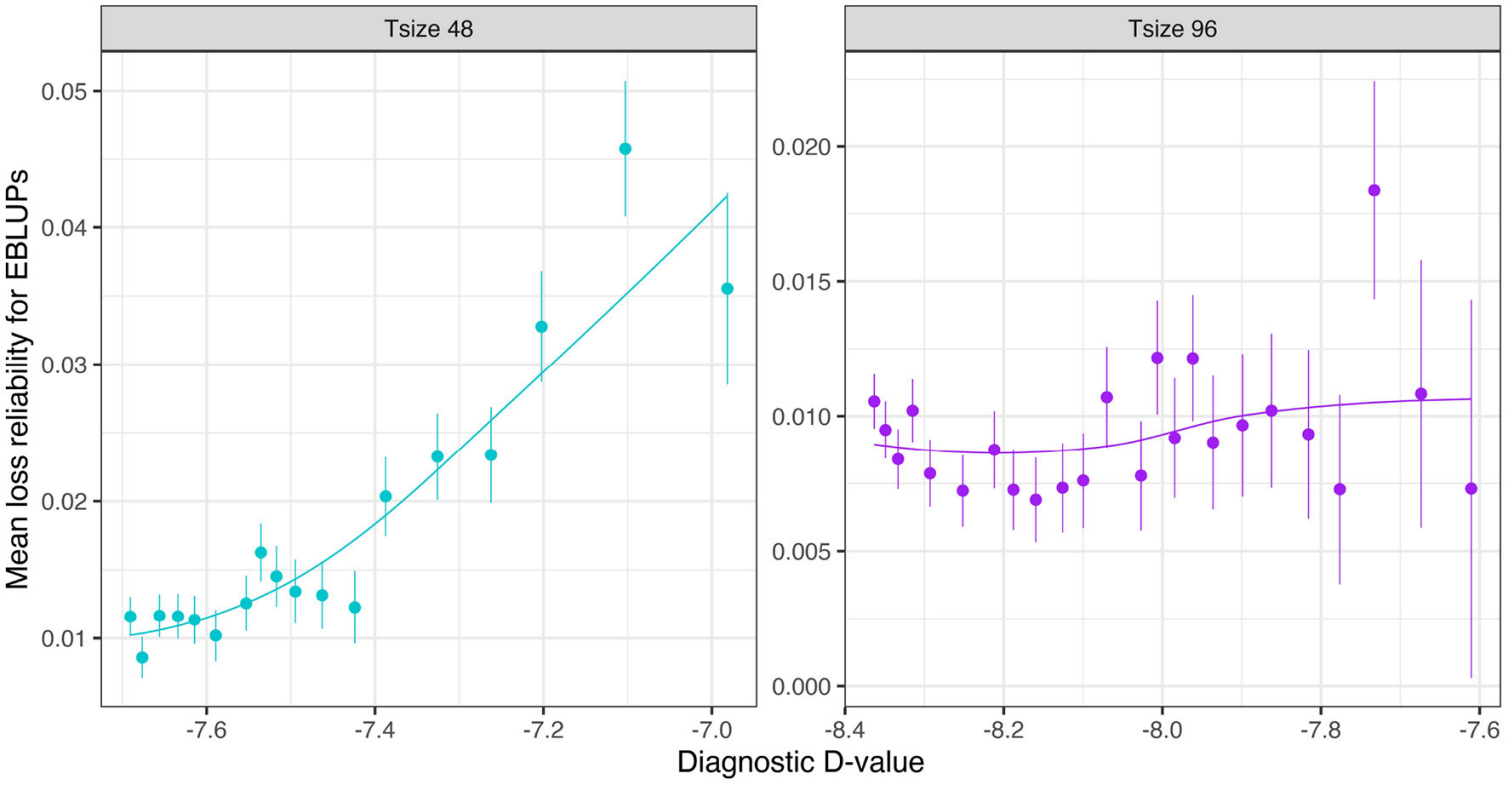
Additive VE effects simulation study: Mean loss in reliability of the EBLUPs of VE effects for Env1 for those varieties that were present in both environments. Each panel corresponds to a different trial size (trials with 48 and 96 varieties) and the points correspond to a sequence of connectivity levels. Also shown are standard errors for each mean (vertical lines) and a loess smoother through the means for each Tsize.

**Figure 11:**
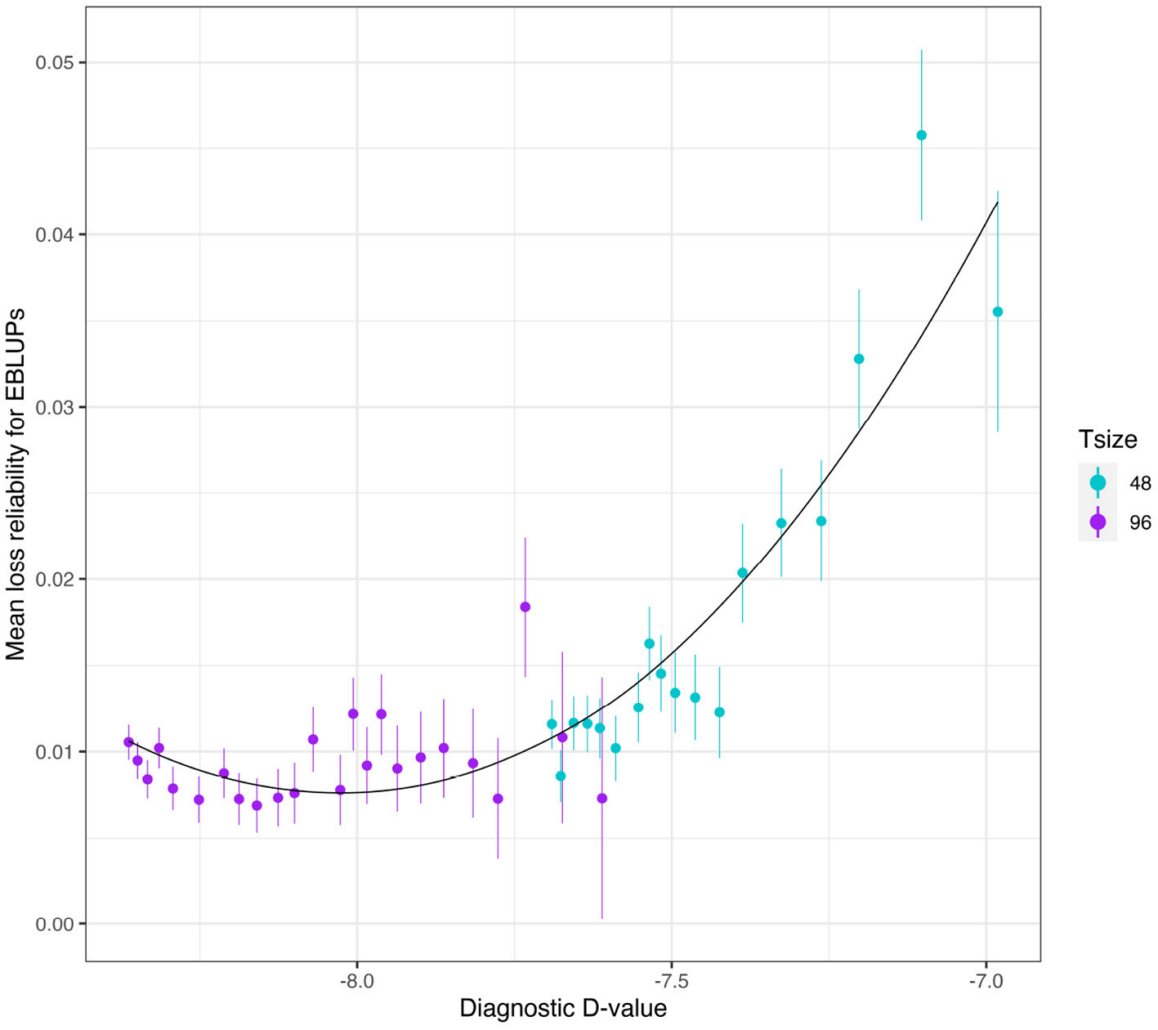
Additive VE effects simulation study: mean loss in reliability of the EBLUPs of VE effects for Env1 for those varieties that were present in both environments. The colours correspond to different trial sizes (trials with 48 and 96 varieties) and the points for each colour correspond to a sequence of connectivity levels. Also shown are standard errors for each mean (vertical lines) and a loess smoother through all the means.

The mean loss in reliability of the EBLUPs of the additive VE effects for Env1 for those varieties that were present in both environments is well predicted by the diagnostic $D_{1c}$(A)-values, both for individual trial sizes (Figure 10) and across trial sizes (Figure 11).

We again investigate the robustness to a change in variance parameters. The same low bound values used in the durum example are used and applied to the same set of simulation studies. These results are provided in Supplementary Materials and highlight similar trends to the results above.

## 4. Discussion

Despite the fact that Multi-environment trials (METs) are a key aspect of plant breeding, there is little in the literature that addresses the “design” of METs in the sense of determining which trials should be combined. Within the paradigm of the factor analytic linear mixed model (FALMM) approach for MET analysis, the aim of both the design and analysis is the reliable prediction of the variety effects for individual environments (VE effects). These are the baseline predictions which can then be summarised across environments in meaningful ways, for example, using the interaction class approach of Smith et al. (2021b).

Smith et al. (2021a) discuss the structure of MET datasets and use a model-based design approach to quantify the amount of information for variety comparisons in a given dataset. They apply the A-optimality measure to variety effects to show the importance of including as many trials as are necessary to capture the selection histories of the varieties under consideration for selection. As in the case of standard model-based design, the calculation of A-values is based on a pre-specified linear mixed model (LMM) so assumes that the associated variance parameters are known. It is important, therefore, to also consider the impact of the structure of a MET dataset on the reliability of genetic variance parameter estimation, as this in turn may affect the reliability of variety predictions, so should be used in conjunction with A-optimality.

In this paper we have developed a diagnostic to be applied to a MET dataset prior to analysis in order to assess the likely reliability of genetic variance parameter estimates, both for the dataset across all environments and for individual environments. As in Smith et al. (2021a) we use a model-based design approach and apply D-optimality measures to genetic variance parameters. Two simulation studies, one using a LMM with independent VE effects and the other additive VE effects, showed that these diagnostic D-values performed well in the sense of predicting the actual reliability of genetic variance parameter estimates.

Historically, variety connectivity between environments was the measure calculated prior to the conduct of a MET analysis to investigate the likely reliability of genetic variance parameter estimation. This measure is simple to compute and intuitively reasonable but there has been nothing in the literature to validate its use. In our simulation studies it was shown that variety connectivity was only able to predict the reliability of genetic variance parameter estimation across connectivity levels for a given trial size (number of varieties in the trial), whereas the new D-optimality diagnostic predicted reliability across both connectivity levels and trial sizes. The application to the real example also suggested that D-optimality encapsulates numerous structural features of a MET data-set that are influential in determining the reliability of genetic variance parameter estimation. These features included, but are not limited to, variety connectivity, trial size and variety replication. Computation of the diagnostic requires specification of variance parameters, namely genetic and residual variances for individual environments and genetic correlations between pairs of environments. Clearly we do not know these values or how they differ between environments prior to the MET analysis. In terms of the latter a pragmatic and sensible approach is to assume homogeneity between environments. The parameter values can be chosen to reflect typical estimates obtained in practise. Both the simulation studies and the real example showed that the diagnostic was robust to the specification of these values.

The pre-specified LMM for the D-optimality diagnostic allowed for the estimation of separate genetic variances for all environments and separate genetic covariances for all pairs of environments. It is therefore targeting MET analyzes that employ factor analytic, or possibly unstructured forms for the genetic variance matrices. It is easy to modify the pre-specified LMM to reflect simpler models, for example, variance component models. Additionally, the partitioning of the genetic effects into additive and non-additive was achieved in this paper using pedigree information but the modification to use genomic (marker) data is straightforward.

The simulation study results suggested that trials with small numbers of varieties will, in general, have larger D-values when compared with trials with more varieties. Even in the case of 100% connectivity, the smallest trial size considered (12 varieties in each of the two trials) resulted in large D-values which then translated to substantial losses in the reliability of VE effect predictions. Additionally, the number of successful model fits was much lower compared with scenario in which there were more varieties in each trial. This is consistent with our experience in analysing MET datasets in which many trials have small numbers of varieties. Even when the connectivity between these and larger trials is high, there are often computational difficulties in fitting the FALMM. In practise, we therefore suggest that individual environment diagnostic values should be examined for a given MET dataset in order to identify environments with large D-values. These environments may contribute insufficient information for genetic variance parameter estimation so their inclusion in the MET dataset should be carefully considered. Additionally, examination of the overall diagnostic value across all environments may be useful. If the overall D-value is large this may indicate insufficient information to fit the gold standard FALMM and it may only be possible to fit a simpler model, such as a variance component model.

## Data Availability Statement

The data analysed in this study is subject to the following licenses/restrictions the datasets presented in this article are not readily available because they are owned by New South Wales Department of Primary Industries and the Grains Research and Development Corporation. Requests to access these datasets should be directed to Gururaj Kadkol, gururaj.kadkol@dpi.nsw.gov.au.

## Author Contributions

CL, BC, and AS conceived the ideas. CL developed and applied the methodology, prepared the data, conducted the simulation study, and prepared the first draft of the manuscript. All authors contributed to the general discussions, manuscript revision, read, and approved the submitted version.

## Funding

CL and AS were supported by the Grains Research and Development Corporation (GRDC) through the Statistics for the Australian Grains Industry (SAGI) project (UW00009).

## Conflict of Interest

The authors declare that the research was conducted in the absence of any commercial or financial relationships that could be construed as a potential conflict of interest.

## Publisher's Note

All claims expressed in this article are solely those of the authors and do not necessarily represent those of their affiliated organizations, or those of the publisher, the editors and the reviewers. Any product that may be evaluated in this article, or claim that may be made by its manufacturer, is not guaranteed or endorsed by the publisher.
