# Supplementary material for: Information Based Diagnostic for Genetic Variance Parameter Estimation in Multi-Environment Trials
Source: Front Plant Sci. 2021 Dec 7;12:785430. doi: 10.3389/fpls.2021.785430 (PMC8688772; doi:10.3389/fpls.2021.785430)
Supplement: Supplementary file 1 [file Data_Sheet_1.pdf]

Supplementary Material

Presented here are the results from a simulation study where we have set the additive genetic variance to 40% of the total genetic variance for each environment and therefore 60% for the non-additive genetic variance, and a between environments correlation of 0.4 for both additive and non-additive Variety by Environment (VE) effects. These values are at the lower end of those seen in practice and are presented here to show the robustness of the diagnostic.

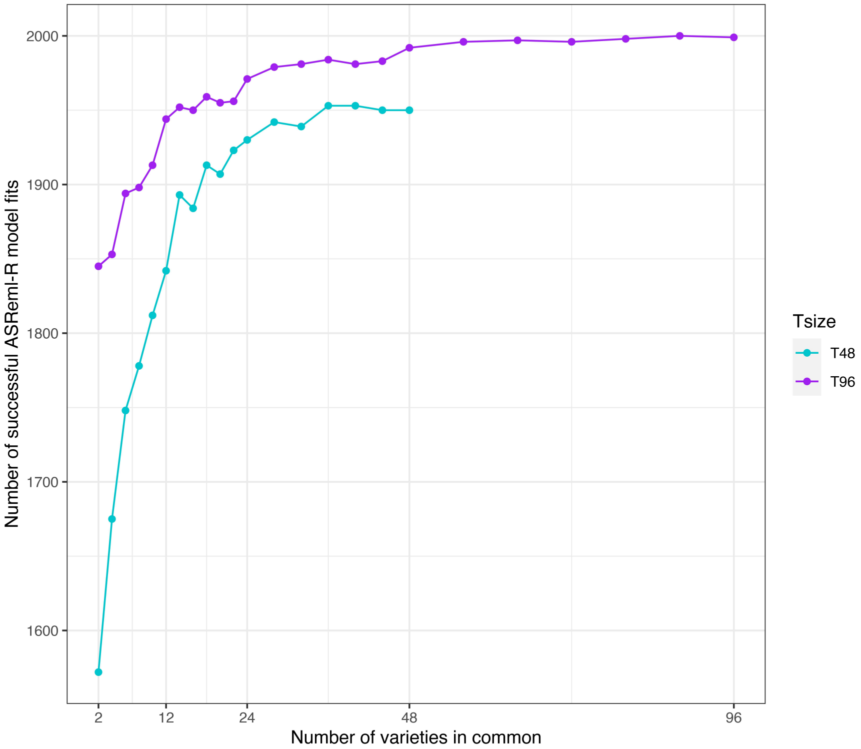

Figure 1: Supplementary low value scenario: Additive VE effects simulation study: number of successful model fits from $N = 2000$ simulations plotted against number of varieties in common for two trial sizes (trials with 48 and 96 varieties). Trial sizes (Tsize) are represented using different colours. Each point within Tsize corresponds to a different level of variety connectivity which ranges from $c = 2$ up to the number of varieties in a trial (representing 100% connectivity between the two trials).

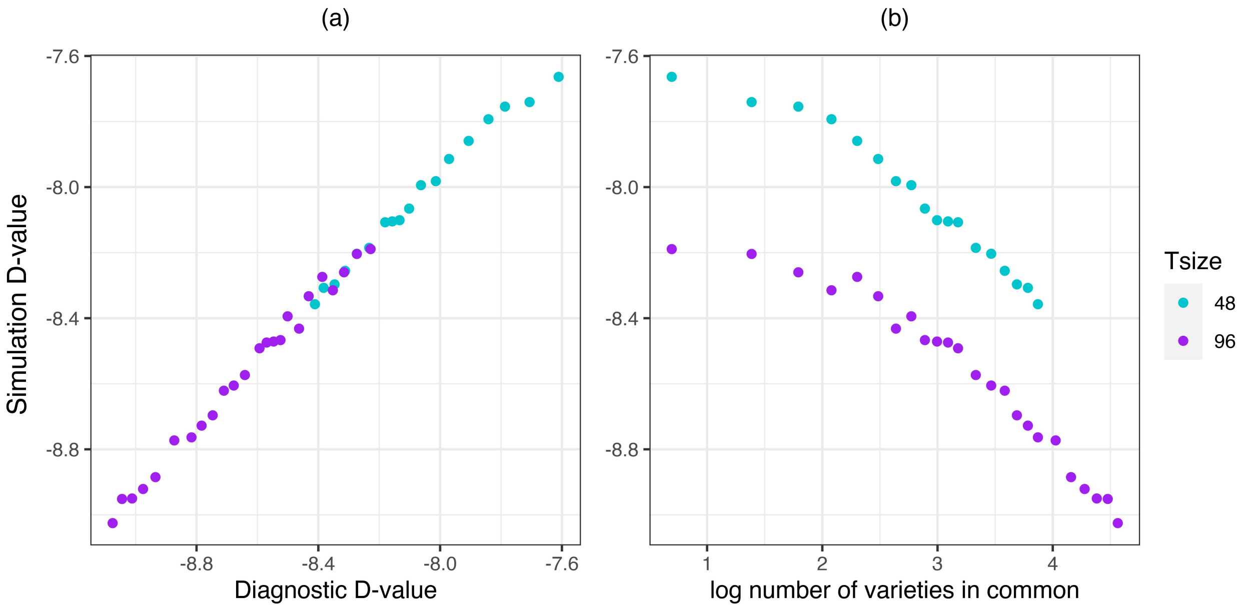

Figure 2: Supplementary low value scenario: Additive VE effects simulation study: simulation based $\mathcal{D}^{s}_{1c}$(A)-values plotted against (a) diagnostic $\mathcal{D}_{1c}$(A)-values and (b) log number of varieties in common for two trial sizes (trials with 48 and 96 varieties) and a sequence of connectivity levels. Trial sizes (Tsize) are represented using different colours. Each point within Tsize corresponds to a different level of variety connectivity which ranges from $c = 2$ up to the number of varieties in a trial (representing 100% connectivity between the two trials).

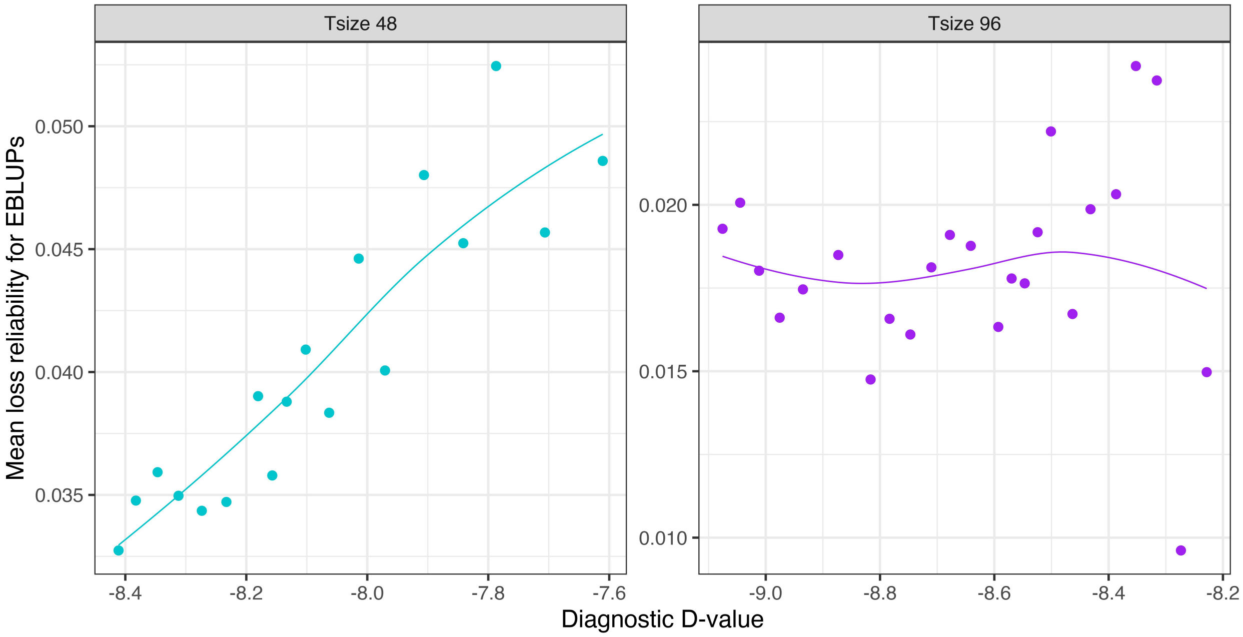

Figure 3: Supplementary low value scenario: Additive VE effects simulation study: mean loss in reliability of the EBLUPs of VE effects for Env1 for those varieties that were present in both environments. Each panel corresponds to a different trial size (trials with 48 and 96 varieties) and the points correspond to a sequence of connectivity levels. Also shown is a loess smoother through the means for each Tsize.

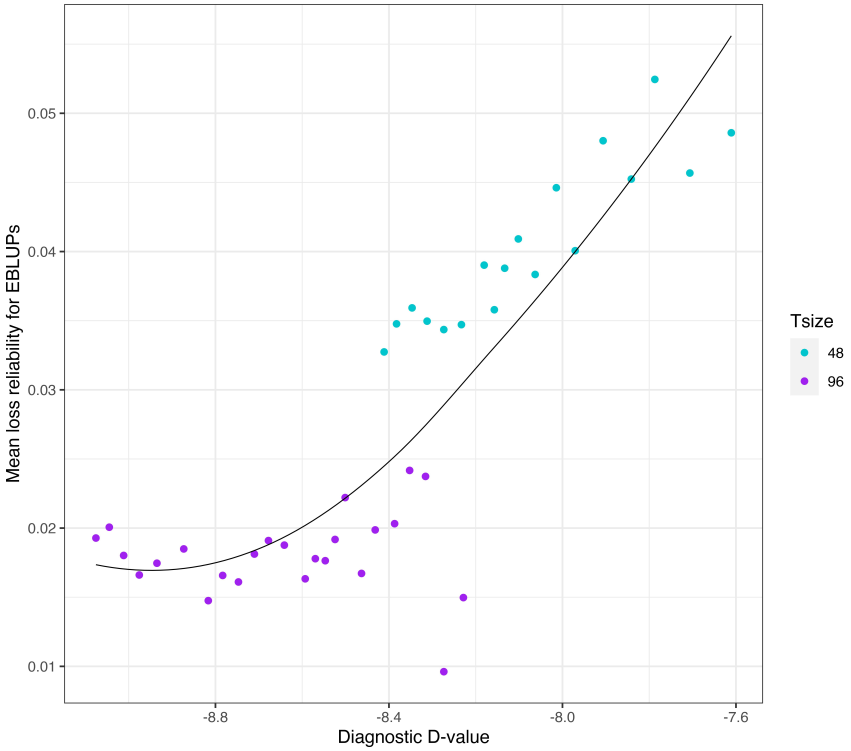

Figure 4: Supplementary low value scenario: Additive VE effects simulation study: mean loss in reliability of the EBLUPs of VE effects for Env1 for those varieties that were present in both environments. The colours correspond to different trial sizes (trials with 48 and 96 varieties) and the points for each colour correspond to a sequence of connectivity levels. Also shown is a loess smoother through all the means.
